# Chemical Signaling Regulates Axon Regeneration via the GPCR–Gqα Pathway in *Caenorhabditis elegans*

**DOI:** 10.1523/JNEUROSCI.0929-21.2021

**Published:** 2022-02-02

**Authors:** Tatsuhiro Shimizu, Kayoko Sugiura, Yoshiki Sakai, Abdul R. Dar, Rebecca A. Butcher, Kunihiro Matsumoto, Naoki Hisamoto

**Affiliations:** ^1^Division of Biological Science, Graduate School of Science, Nagoya University, Nagoya 464-8602, Japan; ^2^Department of Chemistry, University of Florida, Gainesville, Florida 32603

**Keywords:** axon regeneration, *C. elegans*, chemical signaling

## Abstract

Chemical communication controls a wide range of behaviors via conserved signaling networks. Axon regeneration in response to injury is determined by the interaction between the extracellular environment and intrinsic growth potential. In this study, we investigated the role of chemical signaling in axon regeneration in *Caenorhabditis elegans*. We find that the enzymes involved in ascaroside pheromone biosynthesis, ACOX-1.1, ACOX-1.2, and DAF-22, participate in axon regeneration by producing a dauer-inducing ascaroside, ascr#5. We demonstrate that the chemoreceptor genes, *srg-36* and *srg-37*, which encode G-protein-coupled receptors for ascr#5, are required for adult-specific axon regeneration. Furthermore, the activating mutation in *egl-30* encoding Gqα suppresses axon regeneration defective phenotype in *acox-1.1* and *srg-36 srg-37* mutants. Therefore, the ascaroside signaling system provides a unique example of a signaling molecule that regulates the regenerative pathway in the nervous system.

**SIGNIFICANCE STATEMENT** In *Caenorhabditis elegans*, axon regeneration is positively regulated by the EGL-30 Gqα–JNK MAP kinase cascade. However, it remains unclear what signals activate the EGL-30 pathway in axon regeneration. Here, we show that SRG-36 and SRG-37 act as upstream G-protein-coupled receptors (GPCRs) that activate EGL-30. *C. elegans* secretes a family of small-molecule pheromones called ascarosides, which serve various functions in chemical signaling. SRG-36 and SRG-37 are GPCRs for the dauer-inducing ascaroside ascr#5. Consistent with this, we found that ascr#5 activates the axon regeneration pathway via SRG-36/SRG-37 and EGL-30. Thus, ascaroside signaling promotes axon regeneration by activating the GPCR–Gqα pathway.

## Introduction

The ability of axons to regenerate after an injury is a fundamental and conserved property of neurons, which is influenced by the balance between extrinsic factors that inhibit or promote axon outgrowth and the intrinsic growth capacity of neurons (Kaplan et al., 2015). In adult mammals, regeneration following nerve injury occurs efficiently in the peripheral nervous system, whereas the CNS does not successfully regenerate after injury (Mahar and Cavalli, 2018). This difference in regeneration potential has been attributed to the combined effects of extrinsic signals and intrinsic growth capacity (Tedeschi and Bradke, 2017). However, the mechanisms underlying the regulation of regeneration by the extracellular environment in the adult nervous system remain unclear.

**Figure 1.**
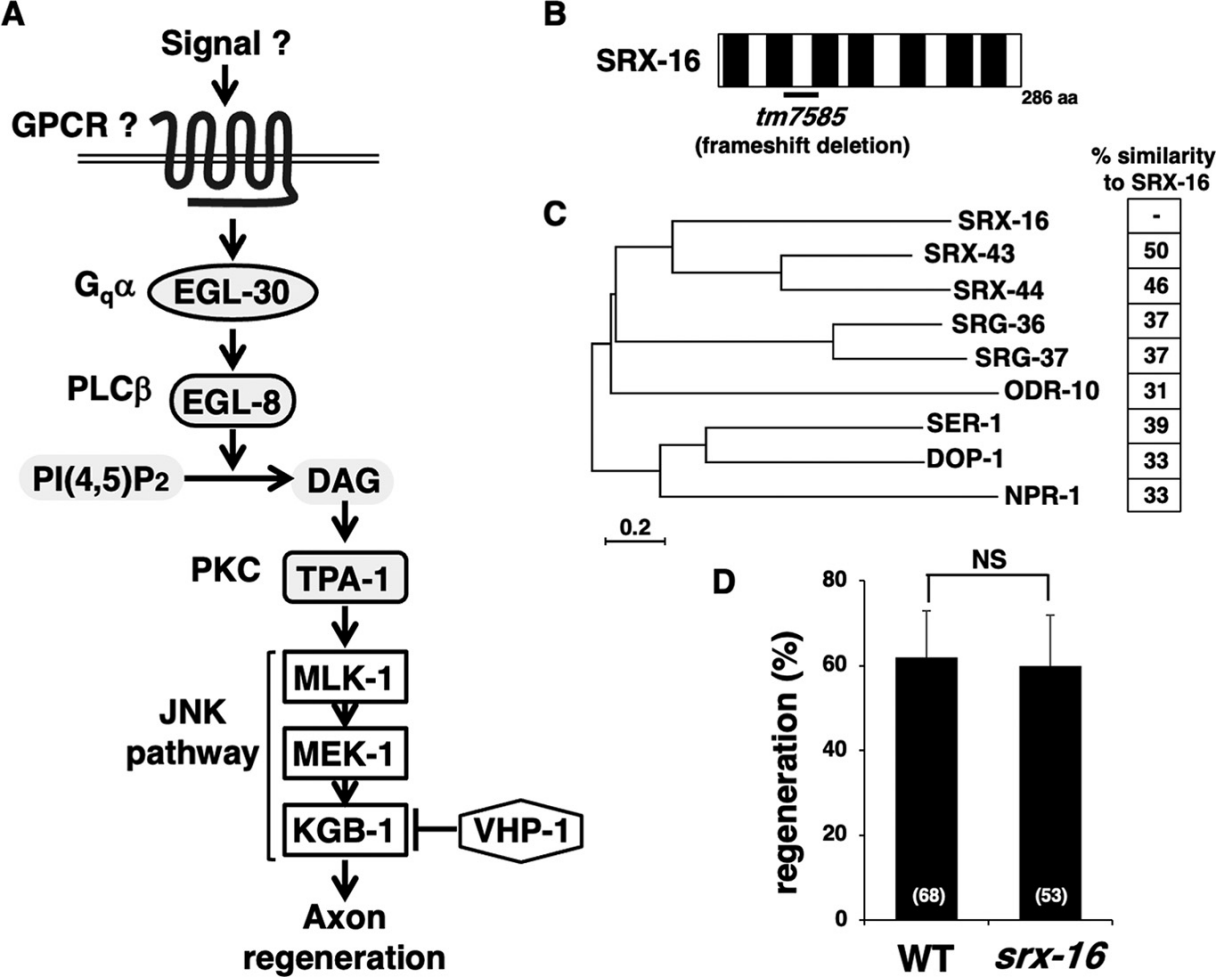
Characterization of SVH-18/SRX-16. ***A***, The EGL-30 pathway regulating axon regeneration. EGL-30 Gqα activates EGL-8 PLCβ, which in turn generates DAG from phosphatidylinositol bisphosphate [PI(4,5)P2]. DAG activates TPA-1 PKC, resulting in activation of the JNK pathway to promote axon regeneration mainly at the young adult stage. The MAPK phosphatase VHP-1 inactivates KGB-1 JNK. ***B***, Structure of SRX-16. The black box indicates the transmembrane regions. The bold line underneath indicates the extent of the deleted region in the *tm7585* mutant. ***C***, Phylogenetic tree depicting the genetic relationships among the GPCRs of *C. elegans*. The phylogenetic tree was constructed using the MEGAX software for Mac. SRX-16 was compared with chemoreceptors of the SRG superfamily and other GPCRs whose ligands have been identified. The scale bar represents the evolutionary distance calculated using the Poisson correction method based on the number of amino acid substitutions per site. The similarity (percentage) of amino acids between SRX-16 and other GPCRs is shown. ***D***, Percentages of axons that initiated regeneration 24 h after laser surgery at the young adult stage. The number of axons examined is shown. Error bars indicate 95% confidence intervals. NS, Not significant.

Recent studies on axon regeneration in the genetic model animal *Caenorhabditis elegans* have revealed that the JNK MAP kinase (MAPK) cascade is a key intrinsic regulator of axon regeneration and may act to sense axonal damage (Fig. 1*A*; Hisamoto and Matsumoto, 2017). The JNK pathway consists of MLK-1 MAPKKK, MEK-1 MAPKK, and KGB-1 JNK; it is inactivated at the KGB-1 activation step by VHP-1, a member of the MAPK phosphatase family (Mizuno et al., 2004). The *vhp-1* loss-of-function (lf) mutation leads to hyperactivation of the JNK pathway, resulting in developmental arrest at an early larval stage. We had previously conducted a genome-wide RNAi screening for suppressors of *vhp-1* lethality and isolated 92 *svh* genes (Li et al., 2012; Shimizu et al., 2021). Analysis of these *svh* genes sheds new light on the regulation of axon regeneration. Moreover, two distinct protein kinases act as MAP4Ks for MLK-1 MAPKKK in a life stage-dependent manner (Pastuhov et al., 2016b). An Ste20-related kinase, MAX-2, phosphorylates and activates MLK-1 primarily at the L4 stage to promote axon regeneration. In contrast, the protein kinase C (PKC) homolog TPA-1 can activate MLK-1 at the young adult stage but not at the L4 stage. The Gqα protein EGL-30 acts as an upstream component of TPA-1. EGL-30 activates a phospholipase Cβ (PLCβ), EGL-8, which in turn generates diacylglycerol (DAG), anactivator of TPA-1 (Fig. 1*A*). Endocannabinoid anandamide (AEA) inhibits axon regeneration via the Goα protein, GOA-1, which antagonizes EGL-30. NPR-19 and NPR-32 function as G-protein-coupled receptors (GPCRs) for AEA (Pastuhov et al., 2016a). Therefore, increased signaling from the AEA pathway suppresses the EGL-30–EGL-8–TPA-1 signaling cascade, which inhibits the activation of the JNK pathway, thereby reducing axon regeneration. However, it remains unclear what signals activate the EGL-30 pathway in the regulation of axon regeneration.

In many organisms, the extracellular environment is interpreted through chemical signaling systems mediated by small molecules (Peso et al., 2015). *C. elegans* secretes a family of small-molecule pheromones called ascarosides, which participate in diverse chemical signaling functions (Park et al., 2019). In particular, ascarosides facilitate the transition to the nonfeeding, long-lived, and highly stress-tolerant dauer stage (Butcher et al., 2007). Ascarosides also have a number of other important effects on behaviors, such as aggregation, avoidance, and mating attraction. Therefore, the animals optimize the extent of these developmental or behavioral decisions by assessing the environmental conditions that affect their survival strategies (Butcher, 2017). Ascarosides are initially synthesized as glycolipids with extremely long side chains that are subsequently shortened by the peroxisomal fatty acid (FA) β-oxidation pathway (Butcher, 2017). This pathway is composed of the following four enzymes: acyl-CoA oxidases (ACOXs), enoyl-CoA hydratase (MAOC-1), hydroxyacyl-CoA dehydrogenase (DHS-28), and β-ketoacyl-CoA thiolase (DAF-22). More than 200 ascaroside-like compounds have been identified by metabolomics, and they are divided into the following two main classes: ω-ascarosides and (ω−1)-ascarosides (von Reuss et al., 2017).

In this study, we found that one dauer-inducing ω-ascaroside, ascr#5 (asc-ωC3; C3), acts on the EGL-30 Gq signaling pathway to promote axon regeneration. Furthermore, we revealed that the *srg-36* and *srg-37* genes, which encode GPCRs for ascr#5, are required for axon regeneration by functioning upstream of EGL-30. These findings strengthen the link between chemical inputs and a conserved regulatory mechanism for axon regeneration.

## Materials and Methods

### *C. elegans* strains.

The *C. elegans* strains used in this study are listed in Table 1. The strains KU501, KU456, and KU457 have been previously reported (Pastuhov et al., 2012). All strains were maintained on nematode growth medium plates and fed with bacteria of the OP50 strain of *Escherichia coli* using the standard method (Brenner, 1974).

**Table 1.** Strains used in this study

Strain	Genotype
KU92	*acox-1.4(km92) I; juIs76 II*
KU501	*juIs76 II*
KU456	*egl-30(ad805) I; juIs76 II*
KU457	*egl-30(tg26) I; juIs76 II*
KU1549	*juIs76 II; srx-16(tm7585) V*
KU1550	*acox-1.1(ok2257) I; juIs76 II*
KU1551	*acox-1.1(ok2257) I; juIs76 II; kmEx1551[Pacox-1.1::acox-1.1* + *Pmyo-2::dsRed-monomer]*
KU1552	*acox-1.1(ok2257) I; juIs76 II; kmEx1552[Pges-1::acox-1.1* + *Pmyo-2::dsRed-monomer]*
KU1553	*acox-1.1(ok2257) I; juIs76 II; kmEx1553[Punc-25::acox-1.1* + *Pmyo-2::dsRed-monomer]*
KU1554	*acox-1.1(ok2257) I; juIs76 II; kmEx1554[Pmec-7::acox-1.1* + *Pmyo-2::dsRed-monomer]*
KU1555	*acox-1.2(gk386052) I; juIs76 II*
KU1556	*acox-1.2(gk386052) I; juIs76 II; kmEx1556[Punc-25::acox-1.2* + *Pmyo-2::dsRed-monomer]*
KU1557	*acox-1.2(gk386052) I; juIs76 II; kmEx1557[Pmec-7::acox-1.2* + *Pmec-7::gfp* + *Pmyo-2::dsRed-monomer]*
KU1558	*acox-1.3(tm5192) I; juIs76 II*
KU1559	*juIs76 II; acox-3 (gk203391) IV*
KU1560	*kmEx1560[Pacox-1.1::nls::gfp::acox-1.1 3'UTR* + *Punc-25::nes::tdTomato* + *Pmyo-2::dsRed-monomer]*
KU1561	*daf-22(ok693) juIs76 II*
KU1562	*daf-22(ok693) juIs76 II; kmEx1562[Punc-25::daf-22* + *Pmyo-2::dsRed-monomer]*
KU1563	*juIs76 II; srg-36 srg-37(kyIR95)X*
KU1564	*juIs76 II; srg-36 srg-37(kyIR95)X; kmEx1564[Psrg-36::srg-36::sl2::gfp* + *Pmyo-2::dsRed-monomer]*
KU1565	*juIs76 II; srg-36 srg-37(kyIR95)X; kmEx1565[Psrg-37::srg-37::sl2::gfp* + *Pmyo-2::dsRed-monomer]*
KU1566	*juIs76 II; srg-36 srg-37(kyIR95)X;* *kmEx1566[Psrg-36::srg-36::sl2::gfp* + *Psrg-37::srg-37::sl2::gfp* + *Pmyo-2::dsRed-monomer]*
KU1567	*egl-30(ad805) I; juIs76 II; srg-36 srg-37 (kyIR95)X*
KU1568	*egl-30(tg26) I; juIs76 II; srg-36 srg-37 (kyIR95)X*
KU1569	*juIs76 II; srg-36 srg-37(kyIR95)X;* *kmEx1569[Punc-25::srg-36::sl2::gfp* + *Psrg-37::srg-37::sl2::gfp* + *Pmyo-2::dsRed-monomer]*
KU1570	*kmEx1570[Psrg-36::srg-36::sl2::gfp* + *Punc-25::nes::tdTomato* + *Pmyo-2::dsRed-monomer]*
KU1571	*acox-1.1(ok2257) egl-30(ad805) I; juIs76 II*
KU1572	*acox-1.1(ok2257) egl-30(tg26) I; juIs76 II*
KU1573	*juIs76 II; srg-36 srg-37(kyIR95)X;* *kmEx1573[Punc-25::srg-36::sl2::gfp* + *Pmyo-2::dsRed-monomer]*
KU1574	*juIs76 II; srg-36 srg-37(kyIR95)X;* *kmEx1574[Punc-25::srg-36::sl2::gfp* + *Punc-25::srg-37::sl2::gfp* + *Pmyo-2::dsRed-monomer]*
KU1575	*juIs76 II; kmEx1557[Pmec-7::acox-1.2* + *Pmec-7::gfp* + *Pmyo-2::dsRed-monomer]*
KU1576	*kmEx1576[Pacox-1.1::nls::gfp::acox-1.1 3'UTR* + *Pmyo-2::dsRed-monomer]*

### Plasmids.

*Pacox-1.1::acox-1.1* was constructed by inserting a genomic DNA, which includes a 1.3 kb region of the *acox-1.1* promoter, the *acox-1.1* coding region, and a 187 bp of the 3′ untranslated region (3′UTR), into pCR2.1. *Pacox-1.1::nls::gfp:: 3*′*UTR (acox-1.1)* was constructed by replacing the *acox-1.1* coding region of the *Pacox-1.1::acox-1.1* plasmid with the nuclear localization signal (NLS)-green fluorescent protein (GFP) region in pPD95.67. The cDNAs used in this study were isolated from the pACT cDNA library (Sakamoto et al., 2005). *Punc-25::acox-1.1* (cDNA), *Punc-25::acox-1.2* (cDNA), and *Punc-25::daf-22* (cDNA) plasmids were constructed by inserting each cDNA into the pSC325 vector. pPD95.75-*Pges-1* (Inoue et al., 2005), pPD52.102, and pPD95.75 vectors were used to construct *Pges-1::acox-1.1*, *Pmec-7::acox-1.1*, *Pmec-7::acox-1.2*, and *Pmec-7::gfp*, respectively. *Psrg-36::srg-36::sl2::gfp* and *Psrg-37::srg-37::sl2::gfp* plasmids (McGrath et al., 2011) are a gift from Cori Bargmann (The Rockefeller University, USA). *Punc-25::srg-36::sl2::gfp* and *Punc-25::srg-37::sl2::gfp* were constructed by replacing the *srg-36* promoter region of *Psrg-36::srg-36::sl2::gfp* and the *srg-37* promoter region of *Psrg-37::srg-37::sl2::gfp* with the *unc-25* promoter of pSC325. *Punc-25::nes::tdTomato* was constructed by replacing the cyan fluorescent protein (CFP) coding region of *Punc-25::nes::cfp* (Hisamoto et al., 2018) with *tdTomato* cDNA (Clontech). *Pmyo-2::dsred-monomer* have been previously described (Li et al., 2012). Promoter regions for the analysis of gene expression patterns were determined by confirming their ability to rescue the phenotype of the corresponding mutant when combined with protein-coding sequences.

### Transgenic animals.

Transgenic animals were obtained using the standard *C. elegans* microinjection method (Table 1; Mello et al., 1991). *Pmyo-2::dsred-monomer*, *Pacox-1.1::acox-1.1*, *Pges-1::acox-1.1*, *Punc-25::acox-1.1*, *Pmec-7::acox-1.1*, *Punc-25::acox-1.2*, *Pmec-7::acox-1.2*, *Pmec-7::gfp*, *Pacox-1.1::nls::gfp*, *Punc-25::daf-22*, *Psrg-36::srg-36::sl2::gfp*, *Psrg-37::srg-37::sl2::gfp*, *Punc-25::srg-36::sl2::gfp*, *Punc-25::srg-37::sl2::gfp*, and *Punc-25::nes::tdTomato* plasmids were used in *kmEx1551* [*Pacox-1.1::acox-1.1* (25 ng/µl) + *Pmyo-2::dsred-monomer* (5 ng/µl)], *kmEx1552* [*Pges-1::acox-1.1* (25 ng/µl) + *Pmyo-2::dsred-monomer* (5 ng/µl)], *kmEx1553* [*Punc-25::acox-1.1* (25 ng/µl) + *Pmyo-2::dsred-monomer* (5 ng/µl)], *kmEx1554* [*Pmec-7::acox-1.1* (25 ng/µl) + *Pmyo-2::dsred-monomer* (5 ng/µl)], *kmEx1556* [*Punc-25::acox-1.2* (25 ng/µl) + *Pmyo-2::dsred-monomer* (5 ng/µl)], *kmEx1557* [*Pmec-7::acox-1.2* (25 ng/µl) + *Pmec-7::gfp* (25 ng/µl) + *Pmyo-2::dsred-monomer* (5 ng/µl)], *kmEx1560* [*Pacox-1.1::nls::gfp* (12.5 ng/µl) + *Punc-25::nes::tdTomato* (25 ng/µl) + *Pmyo-2::dsred-monomer* (5 ng/µl)], *kmEx1576* [*Pacox-1.1::nls::gfp* (12.5 ng/µl) + *Pmyo-2::dsred-monomer* (5 ng/µl)], *kmEx1562* [*Punc-25::daf-22* (25 ng/µl) + *Pmyo-2::dsred-monomer* (5 ng/µl)], *kmEx1564* [*Psrg-36::srg-36::sl2::gfp* (25 ng/µl) + *Pmyo-2::dsred-monomer* (5 ng/µl)], *kmEx1565* [*Psrg-37::srg-37::sl2::gfp* (25 ng/µl) + *Pmyo-2::dsred-monomer* (5 ng/µl)], *kmEx1566* [*Psrg-36::srg-36::sl2::gfp* (25 ng/µl) + *Psrg-37::srg-37::sl2::gfp* (25 ng/µl) + *Pmyo-2::dsred-monomer* (5 ng/µl)], *kmEx1569* [*Punc-25::srg-36::sl2::gfp* (25 ng/µl) + *Psrg-37::srg-37::sl2::gfp* (25 ng/µl) + *Pmyo-2::dsred-monomer* (5 ng/µl)], *kmEx1570* [*Psrg-36::srg-36::sl2::gfp* (25 ng/µl) + *Punc-25::nes::tdTomato* (25 ng/µl) + *Pmyo-2::dsred-monomer* (5 ng/µl)], *kmEx1573* [*Punc-25::srg-36::sl2::gfp* (25 ng/µl) + *Pmyo-2::dsred-monomer* (5 ng/µl)], and *kmEx1574* [*Punc-25::srg-36::sl2::gfp* (25 ng/µl) + *Punc-25::srg-37::sl2::gfp* (25 ng/µl) + *Pmyo-2::dsred-monomer* (5 ng/µl)], respectively.

### Generation of the acox-1.4(km92) mutation using CRISPR–Cas9.

The *acox-1.4(km92)* mutation was generated using the previously described CRISPR–Cas9 system (Dokshin et al., 2018). The CRISPR guide RNA [5′-CCCGUUCCUCGGUGAGAUCCGUUUUAGAGCUAUGCU-3′] was synthesized [Integrated DNA Technologies (IDT)] and coinjected with the transactivating CRISPR RNA (IDT), *Streptococcus pyogenes* Cas9 3NLS (IDT) protein, and the pRF4(*rol-6d*) plasmid into the KU501 strain. Subsequently, each F1 organism carrying the transgene was transferred onto a new dish and used for single-worm PCR, followed by DNA sequencing to detect the mutations. The *acox-1.4(km92)* mutation is a 5 bp deletion in exon 1 of the *acox-1.4* gene, causing a frameshift and premature stop codon in exon 1.

### Microscopy.

Standard fluorescent images of transgenic animals were observed under a 100× objective using a fluorescent microscope (model ECLIPSE E800, Nikon) and photographed using a Zyla CCD camera. Confocal fluorescent images were taken using a confocal laser-scanning microscope (model LSM-800, Zeiss). For analyzing the expression of *acox-1.1* or *srg-36* in GABAergic neurons, >10 axons were analyzed, and gene expression was examined every 30 min for 5 h after injury.

### Axotomy.

Axotomy and microscopy were performed as previously described (Li et al., 2012). Animals were subjected to axotomy at the L4 or young adult stage. The imaged commissures that had growth cones or small branches present on the proximal fragment were counted as “regenerated.” Proximal fragments that showed no change after 24 h were counted as “no regeneration.” A minimum of 15 individuals with 1–3 axotomized commissures were observed for most experiments. Most of the animals with the same genotype regenerated a similar number of axons.

### Pheromone treatment.

ascr#5, synthesized as described previously (Butcher et al., 2008), was dissolved in ethanol and diluted in M9 media containing 0.5% DMSO to a final concentration of 1 μm. After incubating young adult stage animals in this solution for 6 h, axons were cut with a laser and incubated on nematode growth media (NGM) plates containing ascr#5 (1 μm) for 24 h before microscopic observation. For the dauer assay, embryos were incubated on NGM plates containing ascr#5 (1 μm) and the OP50 strain for 3 d at 25°C, and then the numbers of dauer and non-dauer larvae were counted. The rate of dauer formation was calculated by dividing the number of dauer larvae by the total number of dauer and non-dauer larvae.

### Phylogenetic analysis.

Evolutionary relationships among candidate genes were determined by constructing neighbor-joining phylogenetic trees using the MEGAX software (Stecher et al., 2020). Evolutionary distances were calculated using the Poisson correction method (Zuckerkandl and Pauling, 1965).

### Experimental design and statistical analyses.

None of the experiments were randomized, and researchers were not blinded to the group assignments during the experiments and evaluation of results. The sample size was determined based on previous studies that assayed axon regeneration in the GABAergic neurons of *C. elegans* (Yanik et al., 2004; Hammarlund et al., 2009). Approximately 50 axons per animal per group were scored. With this sample size, a 30% difference in axon regeneration was detected with an 80% probability of detection calculated by the Fisher's exact test based on the sample size. However, because of issues (small body size, thin axons, and weak GFP expression for unknown reasons) originating from specific mutants, it was sometimes challenging to excise the same number of axons in all groups. Statistical analysis was conducted as described in a previous study (Pastuhov et al., 2012). Confidence intervals (95%) were calculated using the modified Wald test, and two-tailed *p* values were calculated using Fisher's exact test on GraphPad QuickCalcs (https://www.graphpad.com/quickcalcs/contingency1/). Values with *p* < 0.05 were considered statistically significant.

## Results

### Identification of SVH-18/SRX-16

In *C. elegans*, axon regeneration is regulated by the EGL-30 Gqα–JNK pathway (Fig. 1*A*; Sakai et al., 2021). However, the GPCRs that act upstream of EGL-30 remain unknown. To identify additional components functioning in the JNK pathway, we previously conducted a genome-wide RNAi screening for suppressors of *vhp-1* lethality and isolated 92 *svh* genes (Li et al., 2012; Shimizu et al., 2021). Indeed, we isolated the *egl-30* gene as *svh-12* (Shimizu et al., 2021). To identify GPCRs involved in the EGL-30 signaling pathway, we examined whether *svh* genes encode GPCRs. We identified the *svh-18* gene, which encodes SRX-16, a predicted GPCR chemoreceptor in the *srx* gene family (Fig. 1*B*,C). To determine the effect of *srx-16* on axon regeneration, we assayed regrowth after laser axotomy in GABA-releasing D-type motor neurons, which extend their axons from the ventral to the dorsal nerve cord (Yanik et al., 2004; Hammarlund et al., 2009). In young adult wild-type animals, ∼62% of laser-severed axons could initiate regeneration within 24 h (Fig. 1*D*, Table 2). We found that the *srx-16(tm7585)* deletion mutation (Fig. 1*B*) did not affect axon regeneration (Fig. 1*D*, Table 2). Therefore, the function of SRX-16 in the JNK pathway is to regulate larval growth but not axon regeneration.

**Table 2.** Raw data for genotypes tested by axotomy

Strain	Genotype (*juIs76* background)	Stage	Animals, *n*	Axons, *n*	Regenerations, *n* (% of total)	*p* value	Compared with
KU501*^a^*	wild type	YA	24	68	42 (62%)		
KU1549	*srx-16(tm7585)*	YA	19	53	32 (60%)	0.8493	KU501*^a^*
KU501*^b^*	wild type	YA	24	64	43 (67%)		
KU1550	*acox-1.1(ok2257)*	YA	32	53	22 (42%)	0.0086	KU501*^b^*
KU1551	*acox-1.1(ok2257); Ex[Pacox-1.1::acox-1.1]*	YA	23	52	38 (73%)	0.0015	KU1550
KU456	*egl-30(lf)*	YA	18	50	20 (40%)*		
KU1571	*acox-1.1(ok2257); egl-30(lf)*	YA	23	56	27 (48%)	0.5645	KU1550
KU457	*egl-30(gf)*	YA	13	30	21 (70%)*		
KU1572	*acox-1.1(ok2257); egl-30(gf)*	YA	33	50	34 (68%)	0.0099	KU1550
KU1561	*daf-22(ok693)*	YA	29	50	22 (44%)	0.0217	KU501*^b^*
KU1552	*acox-1.1(ok2257); Ex[Pges-1::acox-1.1]*	YA	36	52	27 (52%)	0.3307	KU1550
KU1553	*acox-1.1(ok2257); Ex[Punc-25::acox-1.1]*	YA	35	51	33 (65%)	0.0200	KU1550
KU1554	*acox-1.1(ok2257); Ex[Pmec-7::acox-1.1]*	YA	19	51	18 (35%)	0.6863	KU1550
KU1562	*daf-22(ok693); Ex[Punc-25::daf-22]*	YA	20	52	34 (65%)	0.0461	KU1561
KU1555*^c^*	*acox-1.2(gk386052)*	YA	35	51	18 (35%)	0.0008	KU501*^b^*
KU1556	*acox-1.2(gk386052); Ex[Punc-25::acox-1.2]*	YA	22	50	30 (60%)	0.017	KU1555*^c^*
KU1558	*acox-1.3(tm5192)*	YA	35	52	27 (52%)	0.1268	KU501*^b^*
KU1559	*acox-3(gk203391)*	YA	32	50	28 (56%)	0.2470	KU501*^b^*
KU92	*acox-1.4(km92)*	YA	21	53	34 (59%)	1	KU501*^b^*
KU1557	*acox-1.2(gk386052);* *Ex[Pmec-7::acox-1.2* + *Pmec-7::gfp]* only D cut	YA	46	46	14 (30%)	0.669	KU1555*^c^*
KU1557	*acox-1.2(gk386052);* *Ex[Pmec-7::acox-1.2* + *Pmec-7::gfp]* PLM+D cut	YA	46	46	30 (65%)	0.0044	KU1555*^c^*
KU1575	*Ex[Pmec-7::acox-1.2* + *Pmec-7::gfp]* only D cut	YA	50	50	35 (70%)	0.4349	KU501*^b^*
KU1575	*Ex[Pmec-7::acox-1.2* + *Pmec-7::gfp]* PLM+D cut	YA	41	41	25 (61%)	1	KU501*^b^*
KU1555*^d^*	*acox-1.2(gk386052)* + EtOH	YA	15	40	13 (33%)		
KU1555	*acox-1.2(gk386052)* + ascr#5 (1 μm)	YA	19	53	34 (64%)	0.0034	KU1555*^d^*
KU1563*^e^*	*srg-36 srg-37(kyIR95)* + EtOH	YA	27	68	28 (41%)		
KU1563	*srg-36 srg-37(kyIR95)* + ascr#5 (1 μm)	YA	22	60	28 (47%)	0.5939	KU1563*^e^*
KU1563*^f^*	*srg-36 srg-37(kyIR95)*	YA	20	50	21 (42%)	0.0083	KU501*^b^*
KU456*^g^*	*egl-30(lf)*	YA	21	54	20 (37%)	0.0016	KU501*^b^*
KU1567	*egl-30(lf) srg-36 srg-37(kyIR95)*	YA	20	50	21 (42%)	1 0.6892	KU1563*^f^* KU456*^g^*
KU1568	*egl-30(gf) srg-36 srg-37(kyIR95)*	YA	28	62	48 (77%)	0.0001	KU1563*^f^*
KU1564	*srg-36 srg-37(kyIR95);* *Ex[Psrg-36::srg-36::sl2::gfp]*	YA	35	92	45 (49%)		
KU1564 Ex(–)	*srg-36 srg-37(kyIR95)*	YA	31	85	31 (36%)	0.1283	KU1564
KU1565	*srg-36 srg-37(kyIR95);* *Ex[Psrg-37::srg-37::sl2::gfp]*	YA	22	63	26 (41%)		
KU1565 Ex(–)	*srg-36 srg-37(kyIR95)*	YA	27	72	24 (33%)	0.3751	KU1565
KU1566	*srg-36 srg-37(kyIR95);* *Ex[Psrg-36::srg-36::sl2::gfp* + *Psrg-37::srg-37::sl2::gfp]*	YA	25	64	36 (56%)		
KU1566 Ex(–)	*srg-36 srg-37(kyIR95)*	YA	29	75	28 (37%)	0.0281	KU1566
KU1569	*srg-36 srg-37(kyIR95);* *Ex[Punc-25::srg-36::sl2::gfp* + *Psrg-37::srg-37::sl2::gfp]*	YA	26	77	42 (55%)		
KU1569 Ex–	*srg-36 srg-37(kyIR95)*	YA	21	63	22 (32%)	0.0281	KU1569
KU1573	*srg-36 srg-37(kyIR95);* *Ex[Punc-25::srg-36::sl2::gfp]*	YA	22	57	19 (33%)		
KU1573 Ex(-)	*srg-36 srg-37(kyIR95)*	YA	22	57	19 (33%)	1	KU1573
KU1574	*srg-36 srg-37(kyIR95);* *Ex[Punc-25::srg-36::sl2::gfp* + *Punc-25::srg-37::sl2::gfp]*	YA	30	81	44 (54%)		
KU1574 Ex(–)	*srg-36 srg-37(kyIR95)*	YA	31	82	27 (36%)	0.0073	KU1574
KU501*^h^*	wild type	L4	24	54	42 (78%)		
KU456	*egl-30(lf)*	L4	15	41	31 (76%)	0.8112	KU501*^h^*
KU1563	*srg-36 srg-37(kyIR95)*	L4	20	52	39 (75%)	0.8206	KU501*^h^*

YA, Young adult.

*a* to *h*: different controls of the same strain.

*Sakai et al. (2021).

### Enzymes involved in ascaroside pheromone biosynthesis participate in axon regeneration

**Figure 2.**
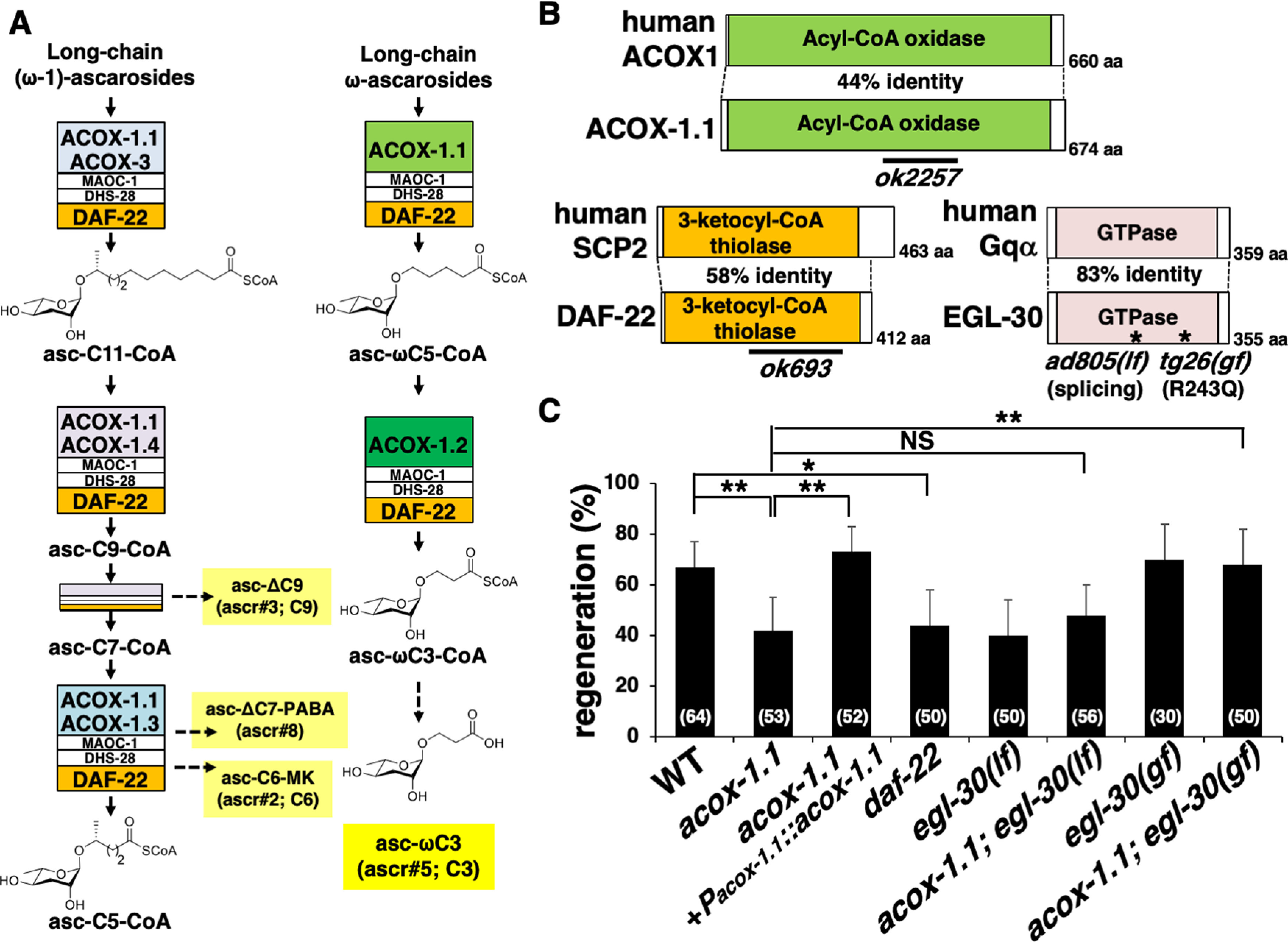
ACOX-1.1 and DAF-22 are required for axon regeneration. ***A***, Ascaroside biosynthesis pathway. Ascarosides fall into two classes—(ω−1)-ascarosides, in which the side chain is attached to the ascarylose sugar at the penultimate (ω−1) position, and ω-ascarosides, in which the side chain is attached at the terminal (ω) position. These two ascaroside classes are shortened by two β-oxidation pathways, one involving ACOX-1.1, ACOX-3, ACOX-1.4, and ACOX-1.3 to produce (ω−1)-ascaroside pheromones (e.g., those shown in yellow boxes) and another involving ACOX-1.1 and ACOX-1.2 to produce ω-ascaroside pheromones (e.g., the one shown in the yellow box). Ascarosides are named based on their structure—asc-(ω)(Δ)C#, with C# indicating the number of carbons in the side chain, ω indicating ω-side chain, and Δ indicating α-β unsaturation. ***B***, Structures of ACOX-1.1, DAF-22, and EGL-30. Schematic domain diagrams of *C. elegans* ACOX-1.1, DAF-22, and EGL-30, and their human counterparts are shown. The regions deleted in *ok2257* and *ok693* are indicated by black bars. The *egl-30* loss-of-function (*ad805*) and gain-of-function (*tg26*) mutations are shown. ***C***, Percentages of axons that initiated regeneration 24 h after laser surgery at the young adult stage. The number of axons examined is shown. Error bars indicate 95% confidence intervals. *p < 0.05, **p < 0.01, as determined by Fisher's exact test. NS, Not significant.

There are 96 members of the SRX family (Robertson and Thomas, 2006). Of those, only SRX-43 and SRX-44 act as GPCRs for the indolated ascaroside, icas#9 (IC-asc-C5; C5; Greene et al., 2016a,b), whereas the functions of the other members are still unknown. The generated phylogenetic tree shows that SRX-16 is similar to SRX-43/SRX-44 (Fig. 1*C*), raising the possibility that the ascaroside pheromone may function as a signal for activating the EGL-30 pathway, which promotes axon regeneration. To test this possibility, we examined whether mutants lacking the enzymes involved in ascaroside production would affect axon regeneration. Ascarosides in *C. elegans* are synthesized by the FA β-oxidation pathway consisting of ACOXs, MAOC-1, DHS-28, and DAF-22 (Fig. 2*A*). We investigated the effects of deletion mutations in *acox-1.1* and *daf-22* (Fig. 2*B*) on the regeneration of D-type motor neurons. The *acox-1.1* gene encodes one of the ACOX enzymes, and the *acox-1.1(ok2257)* deletion has been verified to abolish the ACOX-1.1 function (Fig. 2*A*,*B*; Joo et al., 2010; Zhang et al., 2016, 2018). The *daf-22* gene encodes an ortholog of the human sterol carrier protein SCPx, and the *daf-22(ok693)* deletion results in the loss of its enzymatic function (Fig. 2*A*,*B*; Butcher et al., 2009; Joo et al., 2009). The *acox-1.1(ok2257)* deletion or the *daf-22(ok693)* deficiency causes the accumulation of large fat granules in the intestine, reduced growth rate, and decreased brood size (Joo et al., 2009, 2010). On comparison with wild-type animals, we found that the frequency of axon regeneration after laser axotomy was reduced in *acox-1.1(ok2257)* and *daf-22(ok693)* mutants (Fig. 2*C*, Table 2). To verify that the *acox-1.1* mutation causes this defect in axon regeneration, we generated the transgene *Pacox-1.1::acox-1.1*, which contains the entire genomic *acox-1.1* coding region, its promoter, and the 3′UTR. Introduction of *Pacox-1.1::acox-1.1* into *acox-1.1(ok2257)* mutants significantly rescued the regeneration defect (Fig. 2*C*, Table 2).

To test whether ACOX-1.1 functions in the EGL-30 signaling pathway, we examined the genetic interactions between *acox-1.1* and *egl-30*. EGL-30 is the *C. elegans* Gqα, and we used two *egl-30* alleles, *egl-30(ad805)* and *egl-30(tg26)* (Fig. 2*B*). The *egl-30(ad805)* lf mutation is a mutation in the splice acceptor site that reduces the number of copies of full-length EGL-30 (Brundage et al., 1996). In contrast, the *egl-30(tg26)* gain-of-function (gf) mutation is a missense mutation that constitutively activates EGL-30 function by replacing the conserved Arg-243 with glutamine (Doi and Iwasaki, 2002). We found that the defect in axon regeneration caused by the *acox-1.1(ok2257)* mutation was not enhanced by introducing the *egl-30(lf)* mutation (Fig. 2*C*, Table 2). This result suggests that ACOX-1.1 and EGL-30 act in the same pathway. Moreover, the activating *egl-30(gf)* mutation could suppress the *acox-1.1* phenotype (Fig. 2*C*, Table 2), suggesting that EGL-30 functions downstream of ACOX-1.1. These results support the possibility that ACOX-1.1 regulates axon regeneration through the EGL-30 pathway.

### Expression pattern of *acox-1.1*

**Figure 3.**
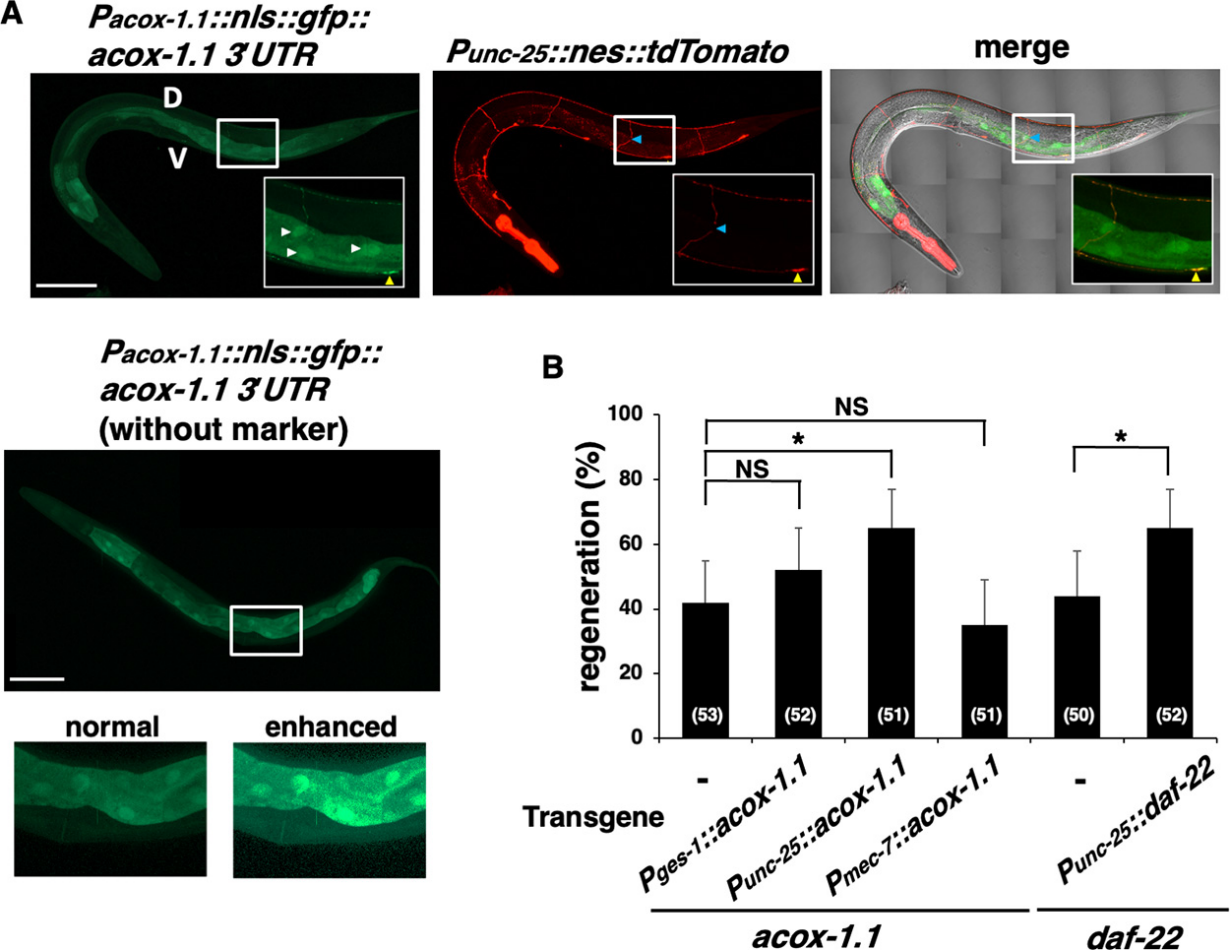
ACOX-1.1 and DAF-22 regulate axon regeneration in a cell-autonomous manner. ***A***, Expression pattern of the *acox-1.1* gene. Fluorescent and differential interference contrast (DIC) images of animals carrying *Pacox-1.1::nls::gfp::acox-1.1 3′UTR* and *Punc-25::nes::tdTomato* 1 h after excision are shown. D-type motor neurons are visualized using tdTomato fused to a nuclear export signal under control of the *unc-25* promoter. Blue, yellow, and white arrowheads indicate a severed axon, the corresponding cell body of the injured neuron, and cell nuclei of gut epithelial cells, respectively. The green signal in D-type motor neurons is absent in the cell nucleus, which may be because of channel bleeding from the strong tdTomato signal. Fluorescent and DIC images of animals carrying only *Pacox-1.1::nls::gfp::acox-1.1 3′UTR* are also shown. The green signal is not seen around the ventral nerve cord where the cell body of GABAergic neurons resides. V, Ventral side; D, dorsal side. Boxed regions are magnified in the insets. Scale bar, 100 µm. ***B***, Percentages of axons that initiated regeneration 24 h after laser surgery at the young adult stage. The number of axons examined is shown. Error bars indicate 95% confidence intervals. *p < 0.05, as determined by Fisher's exact test. NS, Not significant.

To investigate the location of ACOX-1.1 when it regulates axon regeneration, we examined the expression pattern of *acox-1.1*. We constructed a transgene, *Pacox-1.1::nls::gfp::3*′*UTR (acox-1.1)*, which consists of the *acox-1.1* promoter, NLS, GFP, and *acox-1.1* 3′UTR. The *acox-1.1* gene functions in the intestine and hypodermis, where it contributes to the biosynthesis of ascaroside pheromones (Joo et al., 2010). Consistent with this, the *acox-1.1* gene is exclusively expressed in intestinal cells but not in D-type neurons. GFP expression was still not observed in D neurons after axon injury (Fig. 3*A*).

Although *acox-1.1* is expressed in the intestine, we found that the expression of *acox-1.1* cDNA from the *ges-1* promoter in the intestine could not rescue the axon regeneration defect in *acox-1.1(ok2257)* mutants (Fig. 3*B*, Table 2). Recent observations suggest that peroxisomal FA β-oxidation may have an as-yet-unexplored function in neurons (Park and Paik, 2017). Indeed, we found that the *acox-1.1* deficiency was rescued by the expression of *acox-1.1* cDNA from the *unc-25* promoter in D-type motor neurons but not from the *mec-7* promoter in touch neurons (Fig. 3*B*, Table 2). Similar to *acox-1.1*, the expression of *daf-22* with the *unc-25* promoter suppressed the *daf-22* defect (Fig. 3*B*, Table 2). These results suggest that ACOX-1.1 and DAF-22 promote the regeneration of damaged neurons in a cell-autonomous manner.

### Ascaroside ascr#5 promotes axon regeneration

**Figure 4.**
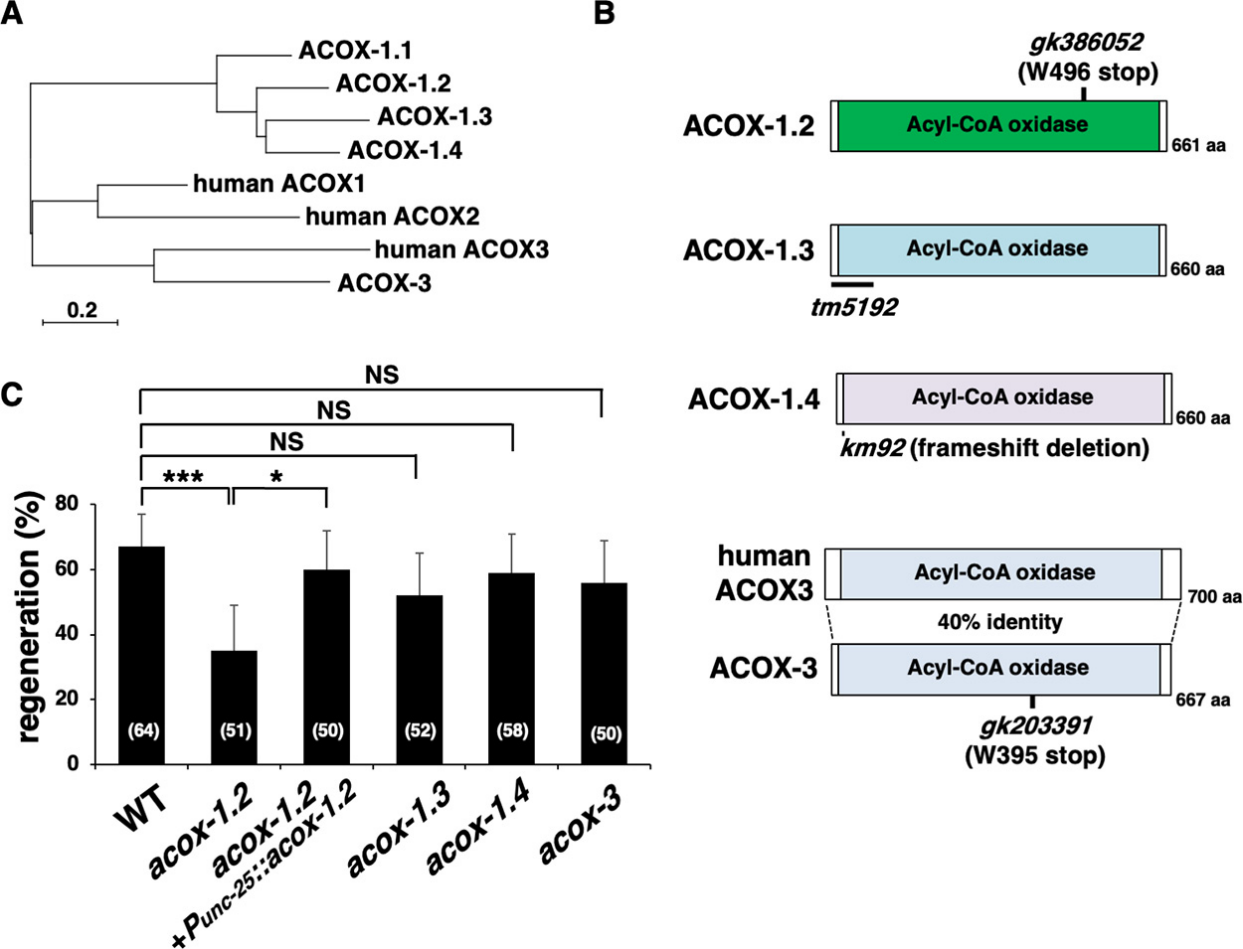
ACOX-1.2 is required for axon regeneration. ***A***, Phylogenetic tree depicting the genetic relationships among ACOX enzymes in *C. elegans* and humans. The phylogenetic tree was constructed using MEGAX software for Mac. The scale bar represents the evolutionary distance calculated using the Poisson correction method based on the number of amino acid substitutions per site. ***B***, Structures of ACOX-1.2, ACOX-1.3, ACOX-1.4, and ACOX-3. Schematic domain diagrams of *C. elegans* ACOX-1.2, ACOX-1.3, ACOX-1.4, and ACOX-3, and human ACOX3 are shown. Additionally, the *gk386052* and *gk203391* mutation sites are indicated. The regions deleted in *tm5192* and *km92* are shown as black bars. ***C***, Percentages of axons that initiated regeneration 24 h after laser surgery at the young adult stage. The number of axons examined is shown. Error bars indicate 95% confidence intervals. *p < 0.05, ***p < 0.001, as determined by Fisher's exact test. NS, Not significant.

We next evaluated which ascaroside regulates axon regeneration. In *C. elegans*, ascarosides are grouped into the following two main classes: ω-ascarosides and (ω−1)-ascarosides (Fig. 2*A*, Butcher, 2017). They are biosynthesized via two parallel β-oxidation pathways, each involving different ACOX enzymes (Fig. 4*A*; Zhang et al., 2015, 2016, 2018). The former pathway involves ACOX-1.2, and the latter depends on ACOX-1.3, ACOX-1.4, and ACOX-3. ACOX-1.1 and DAF-22 are required in both pathways. The *acox-1.2(gk386052)* and *acox-3(gk203391)* mutations contain nonsense mutations, and they are probably null mutations because they result in premature stop codons at Trp-496 and Trp-395, respectively (Fig. 4*B*). We found that the *acox-1.2(gk386052)* mutation reduced axon regeneration (Fig. 4*C*, Table 2). In contrast, the *acox-1.3(tm5192)* deletion, which disrupts the ACOX-1.3 function (Fig. 4*B*, Zhang et al., 2015, 2016) or the *acox-3(gk203391)* mutation had little effect on axon generation (Fig. 4*C*, Table 2). Furthermore, a putative null mutation, *acox-1.4(km92)* (Fig. 4*B*), did not affect axon regeneration (Fig. 4*C*, Table 2). These results suggest that ω-ascarosides are important for axon regeneration.

**Figure 5.**
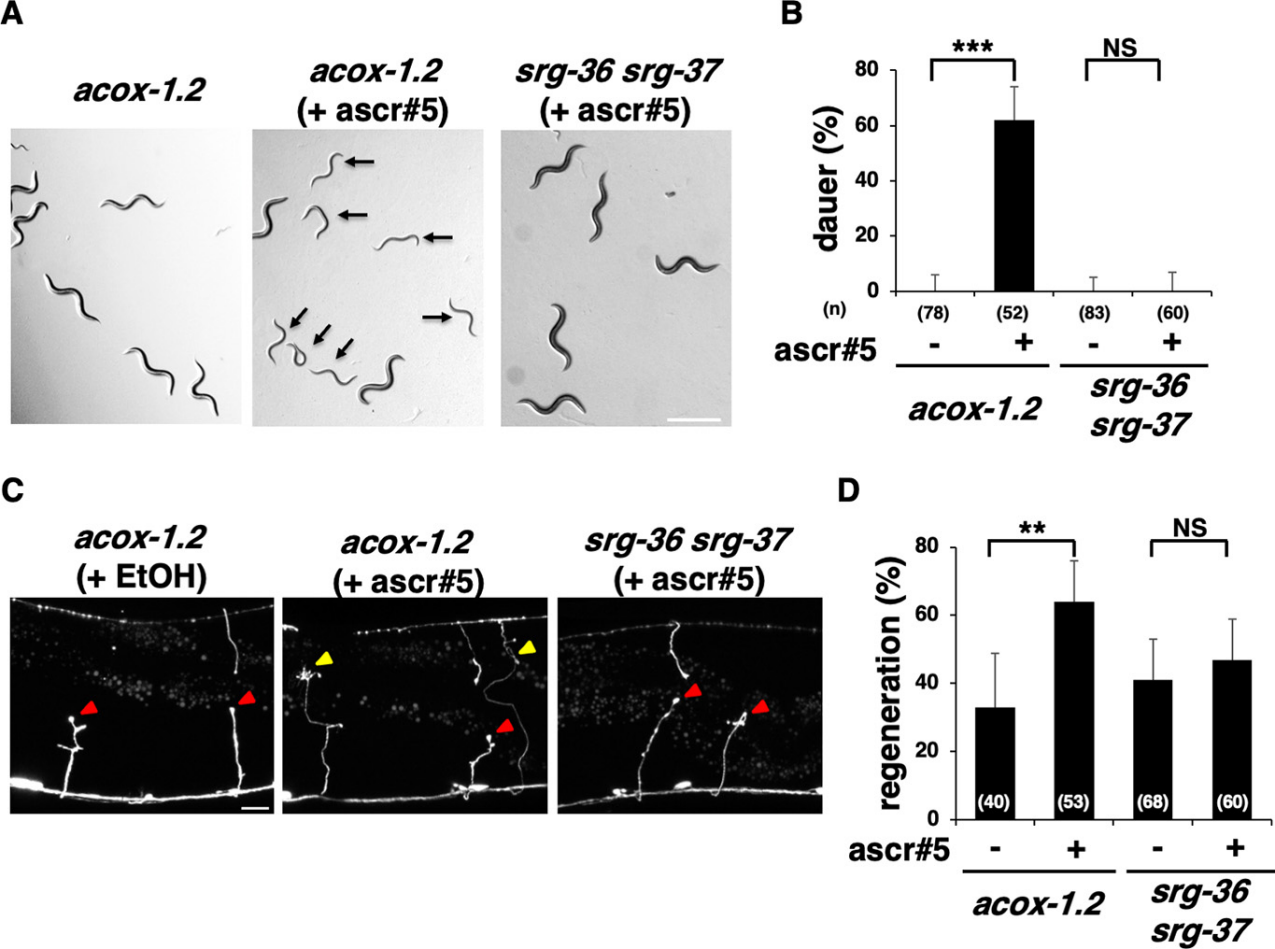
Effects of ascaroside ascr#5 on dauer formation and axon regeneration. ***A***, Dauer induction by ascr#5. Arrows indicate animals entering the dauer stage. Scale bar, 500 µm. ***B***, Percentages of dauer formation. Error bars indicate 95% confidence intervals. ***p < 0.001, as determined by Fisher's exact test. ***C***, Representative D-type motor neurons in animals 24 h after laser surgery. In *acox-1.2* mutants (when ascr#5 was present), severed axons exhibited regenerated growth cones (yellow arrowheads). In *acox-1.2* (when ascr#5 was absent) and *srg-36 srg-37* mutants (when ascr#5 was present), the proximal ends of axons failed to regenerate (red arrowheads). Scale bar, 10 µm. ***D***, Percentages of axons that initiated regeneration 24 h after laser surgery at the young adult stage. The number of axons examined is shown. Error bars indicate 95% confidence intervals. **p < 0.01, as determined by Fisher's exact test. NS, Not significant.

Since ACOX-1.2 influences the production of an ascaroside with a short ω-side chain [i.e., the dauer pheromone asc-ωC3 (C3; ascr#5);& Fig. 2*A*; Zhang et al., 2015], we examined the effect of synthetic ascr#5 on axon regeneration. First, we supplied ascr#5 externally to adult-stage *acox-1.2* mutants and then determined the axon regeneration frequency. We found that the presence of ascr#5 was sufficient to induce dauer formation in *acox-1.2(gk386052)* mutant larvae when added from an embryo (Fig. 5*A*,*B*), and it significantly rescued the axon regeneration defect in *acox-1.2(gk386052)* mutants when introduced at the young adult stage (Fig. 5*C*,*D*, Table 2). These results indicate that ascr#5 is involved in axon regeneration.

### *Acox-1.2* expression inside the injured neuron is required for axon regeneration

**Figure 6.**
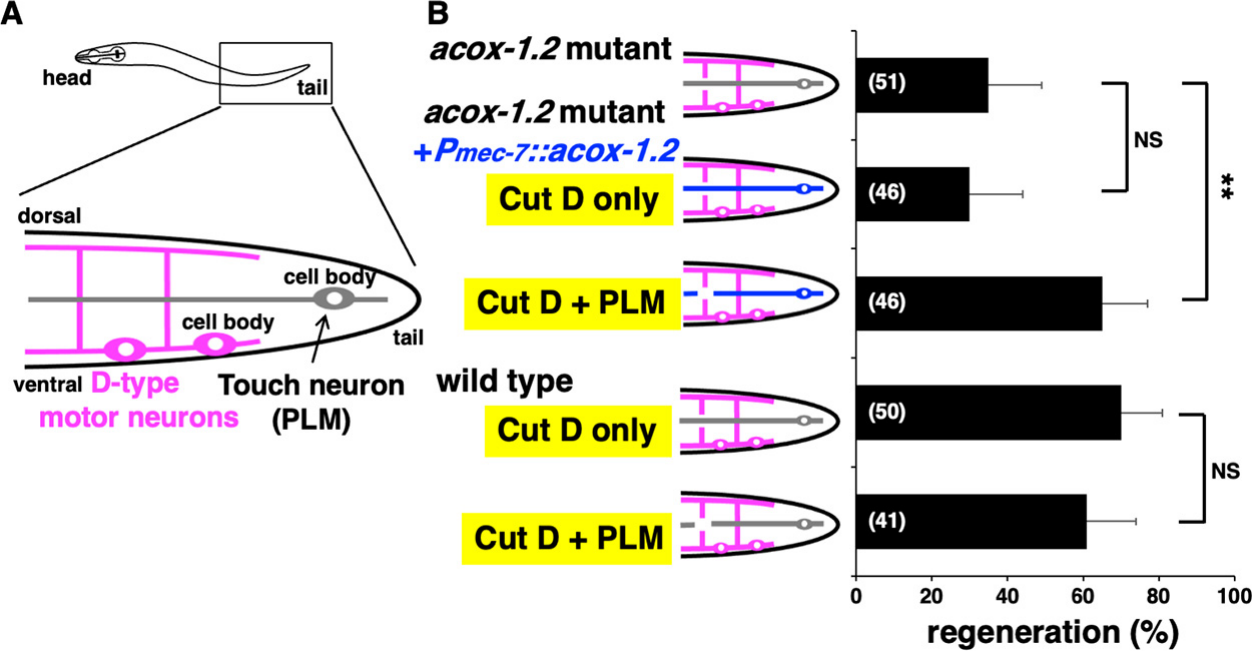
The effect of *acox-1.2* expression in touch neurons on the regeneration of the D-type motor axon. ***A***, Schematic representation of motor and touch neurons. The D-type motor neurons (magenta) have cell bodies on the ventral side and extend axonal commissures dorsally. The touch neuron (gray) extends a long axon parallel to the long body axis and crosses almost perpendicular to the axons of D-type motor neurons. ***B***, Percentages of D-type motor axons that initiated regeneration 24 h after laser surgery in the young adult stage. A schematic representation of motor and touch neurons is shown in the left part. Blue indicates the touch neuron of *acox-1.2* mutants expressing the *acox-1.2* gene. The number of axons examined is shown. Error bars indicate 95% confidence intervals. **p < 0.01, as determined by Fisher's exact test. NS, Not significant.

Expression of *acox-1.2* with the *unc-25* promoter in D neurons suppressed the *acox-1.2* defect (Fig. 4*C*, Table 2), suggesting that ACOX-1.2 regulates axon regeneration in a cell-autonomous manner. Therefore, we examined whether *acox-1.2* expression inside the injured neuron is required for axon regeneration. To test this possibility, we expressed the *acox-1.2* gene in touch neurons using the *mec-7* promoter. Touch neuron axons run parallel to the body axis and intersect perpendicularly to D-type neuron axons (Fig. 6*A*). The expression of *acox-1.2* in touch neurons could not rescue the *acox-1.2* deficiency in D-type motor neuron regeneration (Fig. 6*B*, Table 2). This result is consistent with the possibility that ACOX-1.2 functions cell autonomously in axon regeneration. On the other hand, when both touch and D neurons were severed simultaneously in *acox-1.2(gk386052)* mutants expressing *acox-1.2* in touch neurons, the regeneration defect of D neurons was suppressed (Fig. 6*B*, Table 2). In wild-type animals, simultaneous damage to the axons of touch and D neurons did not affect the frequency of D neuron regeneration (Fig. 6*B*, Table 2). Altogether, these results suggest that ACOX-1.2 induces ascr#5 production in axonally injured touch neurons and that the produced and secreted ascr#5 acts on damaged D neurons to promote regeneration.

### The SRG-36/SRG-37 GPCRs of ascr#5 are involved in axon regeneration

**Figure 7.**
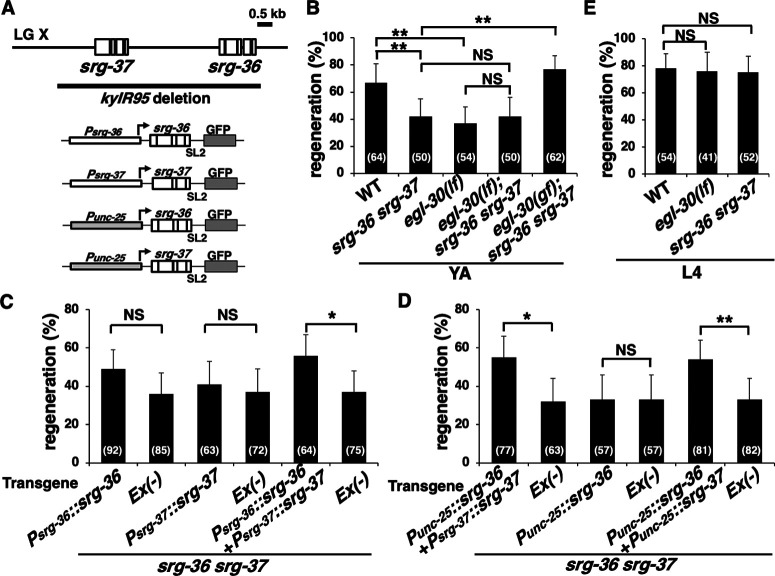
SRG-36 and SRG-37 are involved in axon regeneration. ***A***, Genomic region surrounding *srg-36* and *srg-37*, deletion break points in the *kyIR95* allele, and bicistronic fusion genes. Diagrams for *Psrg-36::srg-36::sl2::gfp*, *Psrg-37::srg-37::sl2::gfp*, *Punc-25::srg-36::sl2::gfp*, and *Punc-25::srg-37::sl2::gfp* are shown in the lower part. ***B*–*D***, Percentages of axons that initiated regeneration 24 h after laser surgery at the young adult (YA) stage. The number of axons examined is shown. Error bars indicate 95% confidence intervals. *p < 0.05, **p < 0.01, as determined by Fisher's exact test. ***E***, Percentages of axons that initiated regeneration 24 h after laser surgery at the L4 stage. The number of axons examined is shown. Error bars indicate 95% confidence intervals. NS, Not significant.

*srg-36* and *srg-37* genes are two members of the nematode-specific GPCR family that encode receptors for ascr#5 (McGrath et al., 2011). We therefore determined whether these GPCRs are involved in axon regeneration. Because the *srg-36* and *srg-37* genes are adjacent to each other in the genome, the *kyIR95* allele deletes both the genes (Fig. 7*A*). We found that at the young adult stage, the frequency of axon regeneration was reduced in *srg-36 srg-37(kyIR95)* (Fig. 7*B*, Table 2). SRG-36 and SRG-37 function redundantly to support dauer formation in response to ascr#5 (McGrath et al., 2011). To investigate whether SRG-36 and SRG-37 also show redundancy in regulating axon regeneration or whether both are required for ascr#5 signaling, the reporter transgenes *Psrg-36::srg-36::sl2::gfp* and *Psrg-37::srg-37::sl2::gfp* (Fig. 7*A*) were introduced into *srg-36 srg-37(kyIR95)* mutants, and we measured the axon regeneration frequency. These transgenes contain bicistronic fusion genes and are functional (McGrath et al., 2011). Transgenic animals expressing either of the two transgenes were defective in axon regeneration, but introducing both transgenes together rescued the *srg-36 srg-37 (kyIR95)* phenotype (Fig. 7*C*, Table 2). These results indicate that both SRG-36 and SRG-37 are required for axon regeneration after laser axotomy.

To confirm that SRG-36/SRG-37 act as receptors for ascr#5 to promote axon regeneration, we examined the effect of ascr#5 addition on axon regeneration in *srg-36 srg-37 (kyIR95)* mutants. As observed previously (McGrath et al., 2011), *srg-36 srg-37 (kyIR95)* mutants were resistant to dauer formation induced by ascr#5 (Fig. 5*A*,*B*). This result is in parallel with the fact that SRG-36/SRG-37 are GPCRs of ascr#5. Similarly, we found that the presence of ascr#5 could not rescue the axon regeneration defect in *srg-36 srg-37 (kyIR95)* mutants (Fig. 5*C*,*D*, Table 2). These results indicate that ascr#5 promotes axon regeneration via SRG-36/SRG-37.

**Figure 8.**
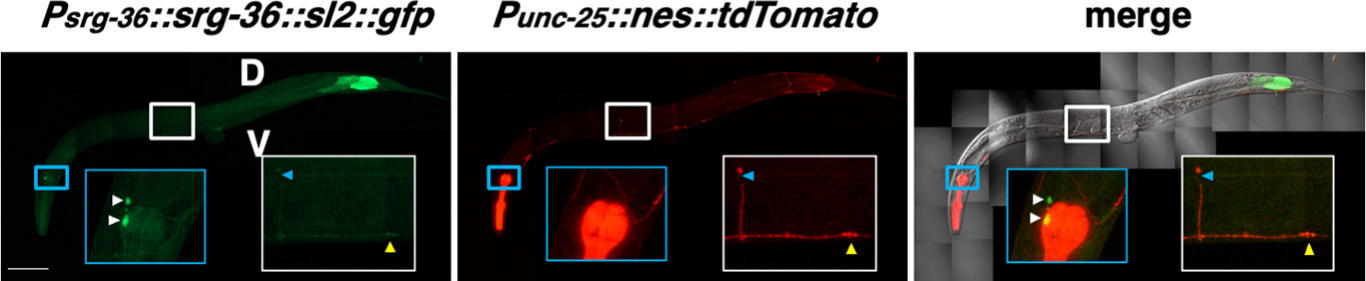
Expression pattern of the *Psrg-36::srg-36::sl2::gfp* gene. Fluorescent and differential interference contrast images of animals carrying *Psrg-36::srg-36::sl2::gfp* and *Punc-25::nes::tdTomato* 1 h after excision are shown. D-type motor neurons are visualized using tdTomato fused to a nuclear export signal under control of the *unc-25* promoter. Blue, yellow, and white arrowheads indicate a severed axon, the corresponding cell body of the injured neuron, and the head neuron ASI, respectively. V, Ventral side; D, dorsal side. Boxed regions are shown magnified in the insets. Scale bar, 100 µm.

To determine whether SRG-36 functions in D-type motor neurons, we examined the expression pattern of *srg-36* using the *Psrg-36::srg-36::sl2::gfp* reporter gene (McGrath et al., 2011). Previous studies have shown that *srg-36* is strongly expressed in ASI neurons but is weakly or inconsistently expressed in several other neurons (McGrath et al., 2011). At the young adult stage, animals carrying *Psrg-36::srg-36::sl2::gfp* did not show GFP expression in D-type motor neurons. In addition, no GFP expression was observed in D neurons after axon injury (Fig. 8). Therefore, to confirm that SRG-36 acts in D-type motor neurons, we expressed *srg-36::sl2::gfp* from the *unc-25* promoter (Fig. 7*A*) in *srg-36 srg-37 (kyIR95)* mutants carrying *Psrg-37::srg-37::sl2::gfp*. We found that the *srg-36* defect in axon regeneration was rescued by the expression of *srg-36* by the *unc-25* promoter in D-type motor neurons (Fig. 7*D*, Table 2). However, the expression of *Punc-25::srg-36::sl2::gfp* alone could not rescue the *srg-36 srg-37 (kyIR95)* mutant phenotype (Fig. 7*D*, Table 2). This is consistent with the idea that both SRG-36 and SRG-37 are required for axon regeneration. Furthermore, the expression of *srg-37::sl2::gfp* from the *unc-25* promoter (Fig. 7*A*) rescued the *srg-36 srg-37 (kyIR95)* mutant phenotype with the *Punc-25::srg-36::sl2::gfp* construct (Fig. 7*D*, Table 2). These results demonstrate that SRG-36 and SRG-37 regulate axon regeneration in injured D-type motor neurons after laser axotomy in a cell-autonomous manner.

### SRG-36 and SRG-37 function in the EGL-30 pathway to promote axon regeneration

Finally, we investigated whether SRG-36/SRG-37 GPCRs function in the EGL-30-mediated pathway to promote axon regeneration. We have previously demonstrated that the CED-10 Rac type GTPase–MAX-2 and EGL-30 Gqα–TPA-1 PKC pathways regulate axon regeneration mainly at the L4 and young adult stages, respectively (Pastuhov et al., 2016b). Furthermore, it has been shown that *max-2* is expressed during early development, but not at the young adult stage (Lucanic et al., 2006). This suggests that TPA-1 replaces MAX-2 to activate MLK-1 during axon regeneration at the adult stage (Fig. 1*A*). Therefore, we examined the relationship between life stage and axon regeneration in *srg-36 srg-37(kyIR95)* mutants. We found that axon regeneration in *srg-36 srg-37(kyIR95)* mutants was reduced only in young adult animals and not in L4 larvae, a phenotype similar to that observed in *egl-30(lf)* mutants (Fig. 7*B*,*E*, Table 2). Thus, SRG-36 and SRG-37 participate in axon regeneration specifically at the adult stage.

We also examined the genetic interactions of *srg-36 srg-37* with *egl-30*. We found that the regeneration defect in *srg-36 srg-37(kyIR95)*; *egl-30(lf)* triple mutants was not greater than the regeneration defect in *srg-36 srg-37(kyIR95)* or *egl-30(lf)* mutants (Fig. 7*B*, Table 2). This result supports the possibility that SRG-36/SRG-37 function in the EGL-30 signaling pathway. Furthermore, the *srg-36 srg-37* phenotype was suppressed by the *egl-30(gf)* mutation (Fig. 7*B*, Table 2), suggesting that SRG-36/SRG-37 function upstream of EGL-30. Thus, SRG-36/SRG-37 GPCRs promote axon regeneration by activating the EGL-30 Gqα pathway.

## Discussion

**Figure 9.**
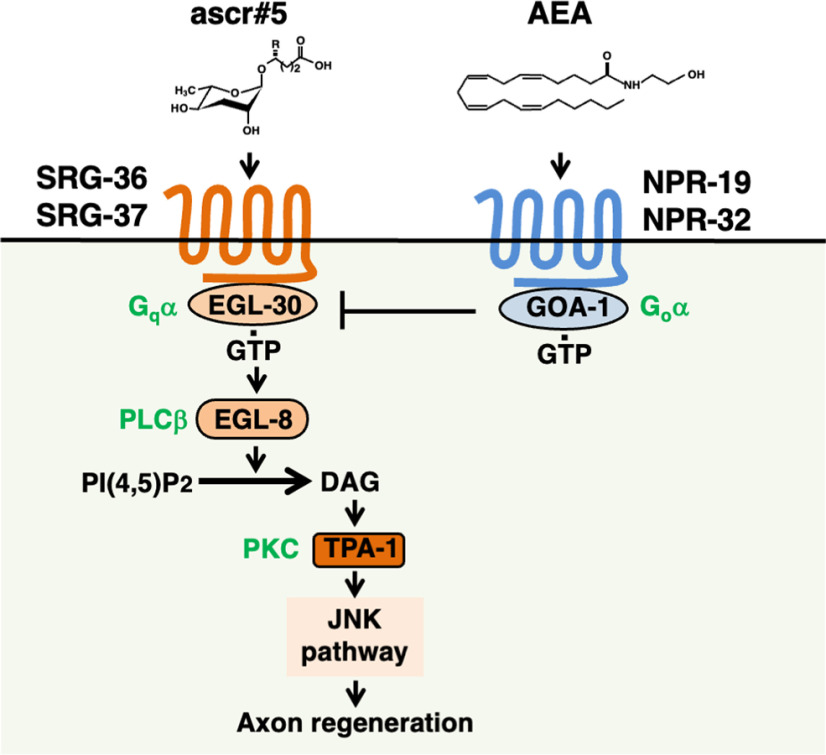
A schematic model for regulating axon regeneration by chemical signaling pathways. ascr#5 activates the EGL-30–EGL-8–TPA-1 signaling cascade via SRG-36/SRG-37 GPCRs to promote axon regeneration. AEA activates the Goα protein GOA-1 via NPR-19/NPR-32 GPCRs, which antagonizes EGL-30, inhibiting axon regeneration. Axon regeneration in *C. elegans* is determined by the balance of stimulatory (ascr#5) and inhibitory (AEA) chemical signals transduced by Gα protein signaling pathways.

Pheromones are molecules secreted by individuals that can induce changes in the behavior and development of different animals of the same species. *C. elegans* secretes ascarosides, which constitute a conserved family of signaling molecules, as pheromones to communicate with other animals and to coordinate population development and behavior (Peso et al., 2015). Originally, ascarosides were identified as components of the dauer pheromone, which is the population density signal. High population density results in ascaroside accumulation, which in combination with additional environmental stimuli, such as limited food availability and temperature stress, promotes larval arrest in the dauer stage (Golden and Riddle, 1984). In this study, we found that ascaroside signaling regulates neural processes in *C*. *elegans*. In particular, we show that the loss of ascaroside production impairs axon regeneration. Furthermore, GPCR sensing of ascaroside regulates axon regeneration via the EGL-30 Gqα signaling pathway (Fig. 9).

The primary site of ascaroside biosynthesis appears to be the intestine, and ascaroside is also likely to be excreted via the intestine (Butcher et al., 2009). However, the expression of the *acox-1.1* gene in the intestine fails to rescue the axon regeneration defect in *acox-1.1* mutants, whereas its expression in injured neurons can restore the *acox-1.1* deficiency. Thus, ACOX-1.1 promotes the regeneration of damaged neurons in a cell-autonomous manner. Furthermore, we demonstrated that *acox-1.2* expression in injured neurons has an important function in regulating axon regeneration. Expression of *acox-1.2* in touch neurons does not rescue the *acox-1.2* deficiency in D-type motor neuron regeneration. However, in *acox-1.2* mutants expressing *acox-1.2* in touch neurons, simultaneous laser ablation of axons of D and touch neurons rescues the regeneration defect of D neurons. These results suggest that ACOX-1.2 expressed in the damaged touch neuron induces the production of ascaroside, which in turn acts on the nearby damaged D neuron to induce regeneration. Thus, ascaroside is synthesized in axotomized neurons and is required for axon regeneration, suggesting that ascaroside regulates axon regeneration as a nonpheromone signal. Since axotomized neurons gain the ability to synthesize ascaroside in response to axon injury, it appears that transcriptional regulation is necessary to ensure that sufficient amounts of ascaroside are available when axons are damaged. Therefore, it is important to identify the transcription factors that regulate the transcription of genes for ascaroside synthesis after axon injury. Since our *svh* screening revealed genes that encode transcription factors (Shimizu et al., 2021), analysis of these *svh* genes will shed new light into the mechanism underlying the transcription of ascaroside synthesis genes regulated in response to axon injury.

What is the ascaroside that regulates axon regeneration? We show that mutations in *acox-1.1*, *daf-22*, and *acox-1.2* are defective in axon regeneration, whereas *acox-3*, *acox-1.3*, or *acox-1.4* mutations have little effect on axon regeneration. ACOX-1.1, DAF-22, and ACOX-1.2 participate in the β-oxidation cycles that shorten ω-ascarosides, with ACOX-1.2 specifically participating in the last of these β-oxidation cycles, which generates ascr#5 (Zhang et al., 2018). An *acox-1.2* mutant only shows defects in the production of ascr#5 and not in that of other ascarosides. Thus, ascr#5 is a candidate ascaroside responsible for promoting axon regeneration after axon injury. Indeed, ascr#5 recovers the ability of the *acox-1.2* mutant to regenerate axons. The ascaroside signal is sensed by GPCRs in specific chemosensory neurons (Butcher, 2017). *srg-36* and *srg-37* genes encode GPCRs for ascr#5 (McGrath et al., 2011). We found that the *srg-36 srg-37* double mutation decreases axon regeneration ability. This indicates that *C. elegans* uses SRG-36/SRG-37 GPCRs to recognize ascr#5 for initiating axon regeneration after axon injury. The expression of GPCRs for sensing ascarosides is clearly predominant in sensory neurons, whereas *srg-36*/*srg-37* function in injured motor neurons. In most cases, each GPCR is strictly expressed in different cell types, contributing to their distinct and cell type-specific responses to internal signals (Rohrer and Kobilka, 1998). Furthermore, recent studies have revealed that the expression of several chemoreceptor genes, such as *srh-234* and *odr-10*, is regulated by environmental stimuli (Gruner et al., 2014; Ryan et al., 2014). These results raise the possibility that SRG-36 and SRG-37 are produced in response to nerve injury, activating ascr#5 signaling via the autocrine system and inducing axon regeneration.

The ascr#5-specific SRG-36 and SRG-37 provide an example for highly structure-specific ascaroside receptors (McGrath et al., 2011). Our finding that SRG-36 and SRG-37 do not function redundantly in axon regeneration suggests that heterodimerization of SRG-36 and SRG-37 may be necessary to form a functional complex for signal transduction. Recent studies have suggested that GPCRs associate as dimers or higher-order oligomers (Lause, 2010). For example, SRBC-64/SRBC-66 function as part of receptor GPCR dimers or higher-order oligomers, including more specific receptors, such as DAF-37 (Park et al., 2012). Thus, the complex ascaroside signaling properties may partly be due the interaction of several different ascaroside receptors that bind directly to ascarosides.

Although ascarosides are specific to nematodes, other similar lipid molecules may contribute to promoting axon regeneration in mammals. Peroxisome proliferator-activated receptor α (PPARα) induces ACOX gene expression in mammals (Marcus et al., 1993). In rat dorsal root ganglion neurons, axonal damage increases PPAR protein levels, causing PPAR transport from the distal axons to the nucleus and promoting neuronal regeneration (Lezana et al., 2016). Furthermore, thiazolidinedione, a PPAR agonist, promotes axonal growth in rat hippocampal neurons by activating the JNK pathway in a PPARα-dependent manner (Quintanilla et al., 2013). Therefore, it is possible that lipid metabolites produced by ACOX enzymes in the peroxisome may induce JNK activation and promote axon regeneration.

Each GPCR couples preferentially with a functionally distinct class of Gα proteins (Möller et al., 2001). In this study, we found that SRG-36/SRG-37 GPCRs activate EGL-30 Gqα and promote axon regeneration. We have recently demonstrated that AEA modulates the axon regeneration response of GABAergic motor neurons after laser axotomy (Pastuhov et al., 2012, 2016a). AEA functions as an inhibitory signal for axon regeneration, which is transmitted through the NPR-19/NPR-32 GPCR–GOA-1 Goα pathway and antagonizes EGL-30 Gqα. Therefore, axon regeneration in *C. elegans* appears to be determined by the balance of stimulatory and inhibitory signals, such as ascaroside and AEA, which are transduced by Gα protein signal transduction pathways (Fig. 9). Thus, axon regeneration after axonal injury in *C. elegans* is regulated by the G_o_α–G_q_α signaling pathway.
